# Supplementary material for: Design, Synthesis and Antibacterial Evaluation of 3-Substituted Ocotillol-Type Derivatives
Source: Molecules. 2018 Dec 14;23(12):3320. doi: 10.3390/molecules23123320 (PMC6321515; doi:10.3390/molecules23123320)
Supplement: Supplementary file 1 [file molecules-23-03320-s001.pdf]

Supplementary Materials

Design, Synthesis and Antibacterial Evaluation of 3-Substituted Ocotillol-Type Derivatives

Kai-Yi Wang [1], Zhi-Wen Zhou [2], Heng-Yuan Zhang [2], Yu-Cheng Cao [1], Jin-Yi Xu [2], Cong Ma [3], Qing-Guo Meng [1,*] and Yi Bi [1,*]

[1] School of Pharmacy, Key Laboratory of Molecular Pharmacology and Drug Evaluation, Ministry of Education, Collaborative Innovation Center of Advanced Drug Delivery System and Biotech Drugs in Universities of Shandong, Yantai University, Yantai, 264005, China; wangky_1994@163.com (K.-Y.W.); cchm1187829@163.com (Y.-C.C.)

[2] State Key Laboratory of Natural Medicines and Department of Medicinal Chemistry, China Pharmaceutical University, Nanjing, 210009, China; 18694088106@163.com (Z.-W.Z.); panacea0928@163.com (H.-Y.Z.); jinyixu@china.com (J.-Y.X.)

[3] Department of Applied Biology and Chemical Technology, and State Key Laboratory of Chirosciences, The Hong Kong Polytechnic University, Hung Hom, Kowloon, Hong Kong, China; cong.ma@polyu.edu.hk

* Correspondence: beeyee_413@163.com (Y.B.); qinggmeng@163.com (Q.-G.M.); Tel.: +86-0535-6706285 (Y.B.); +86-0535-6706022 (Q.-G.M.)

Received: 30 November 2018; Accepted: 13 December 2018; Published: 14 December 2018

Table of Contents

Copies of ^{1}H-NMR, ^{13}C-NMR and HR-MS (ESI) spectra ....Pages S2-S21

Copies of ^{1}H NMR, ^{13}C NMR and HR-MS (ESI) spectra

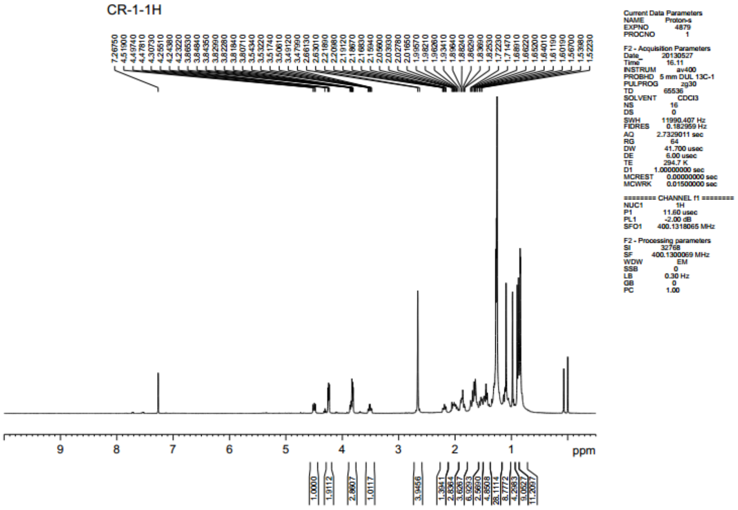

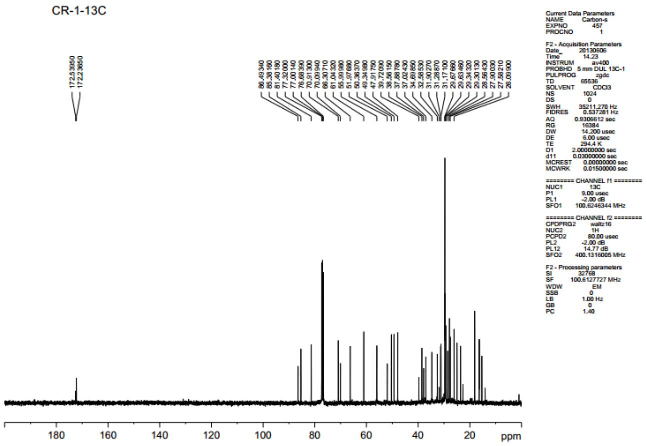

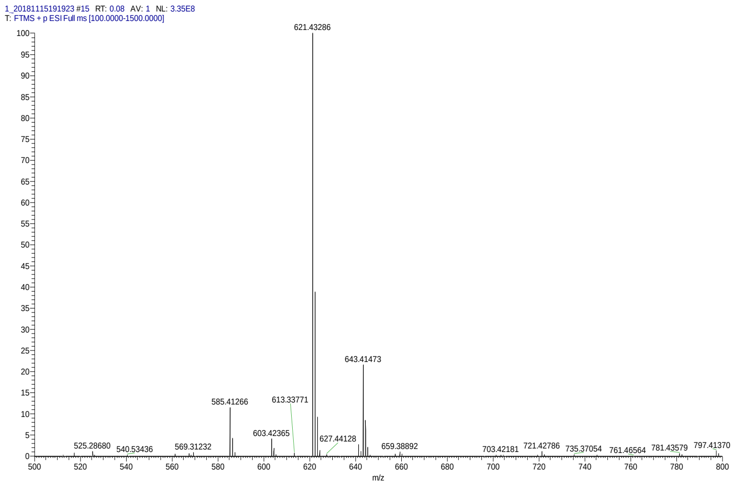

^{1}H NMR, ^{13}C NMR and HR-MS (ESI) spectra of compound **7**

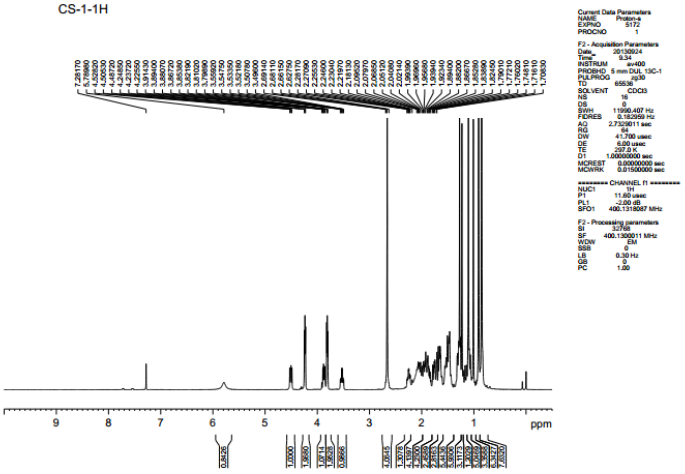

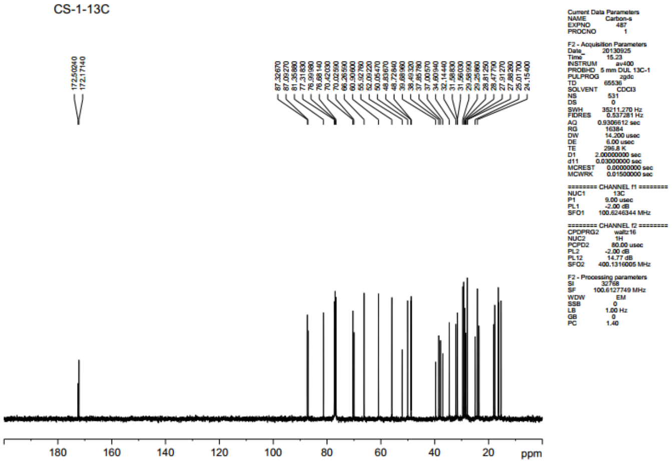

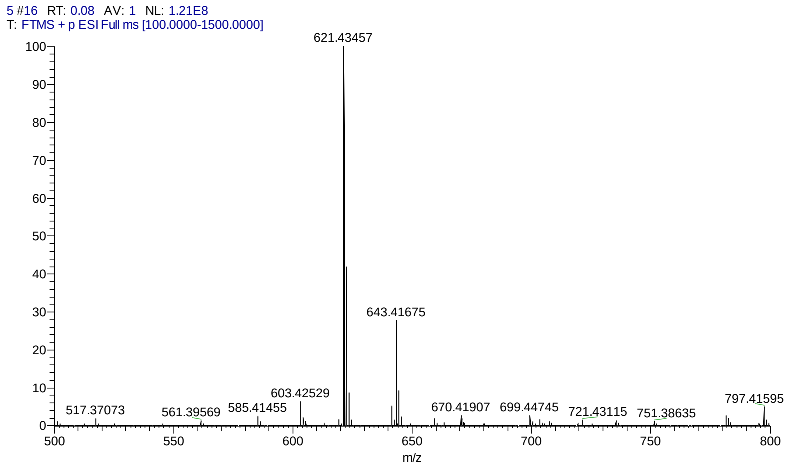

^{1}H NMR, ^{13}C NMR and HR-MS (ESI) spectra of compound **8**

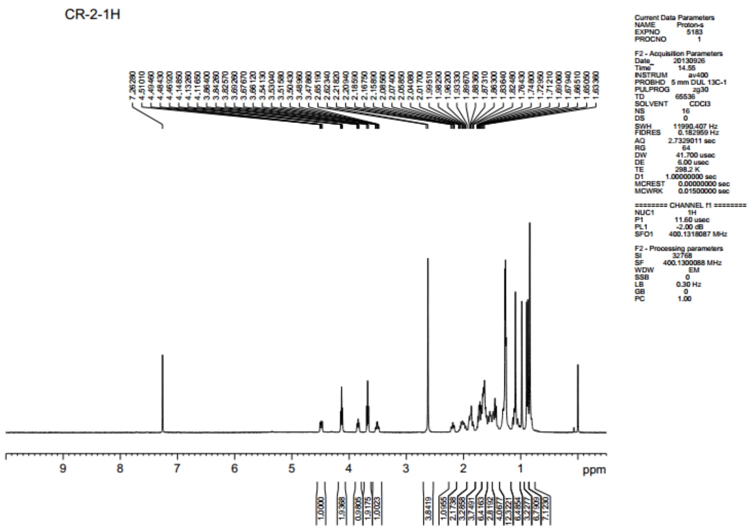

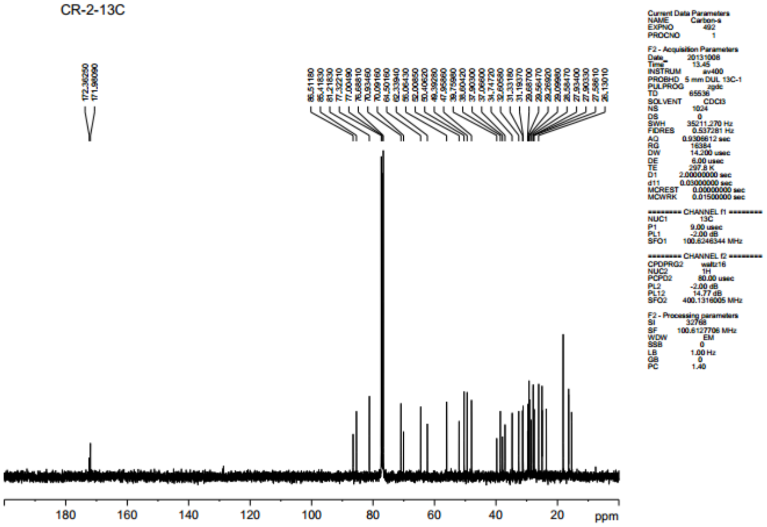

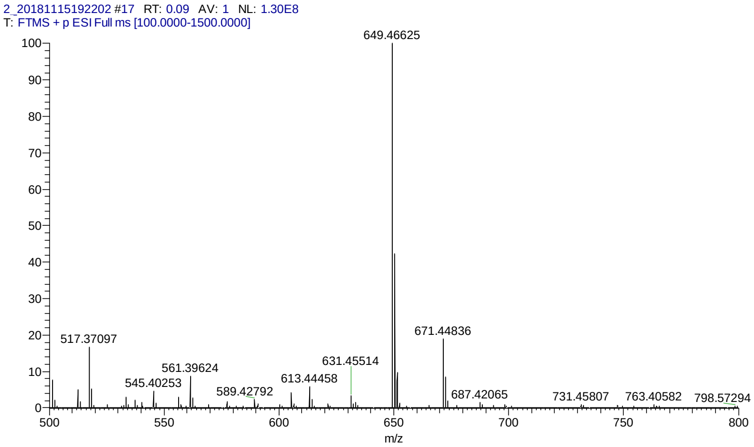

^{1}H NMR, ^{13}C NMR and HR-MS (ESI) spectra of compound **9**

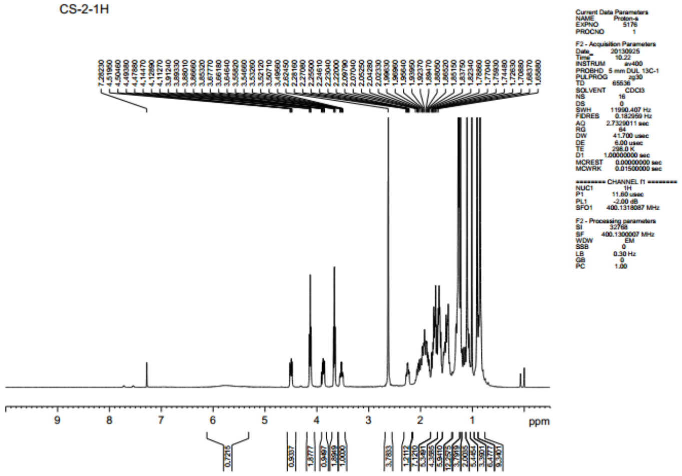

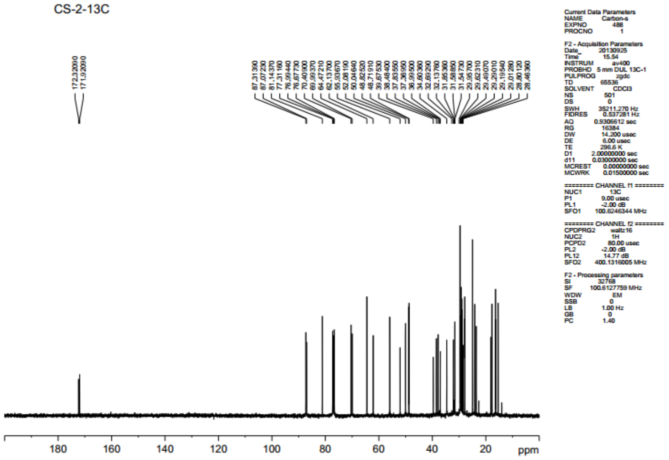

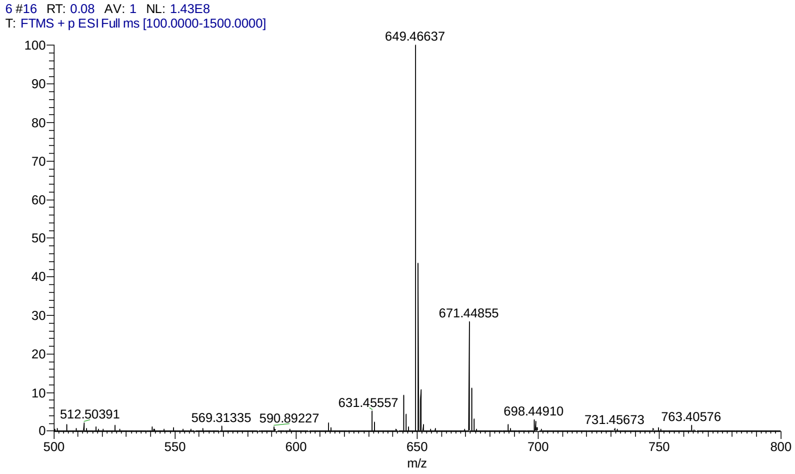

¹H NMR, ¹³C NMR and HR-MS(ESI) spectra of compound **10**

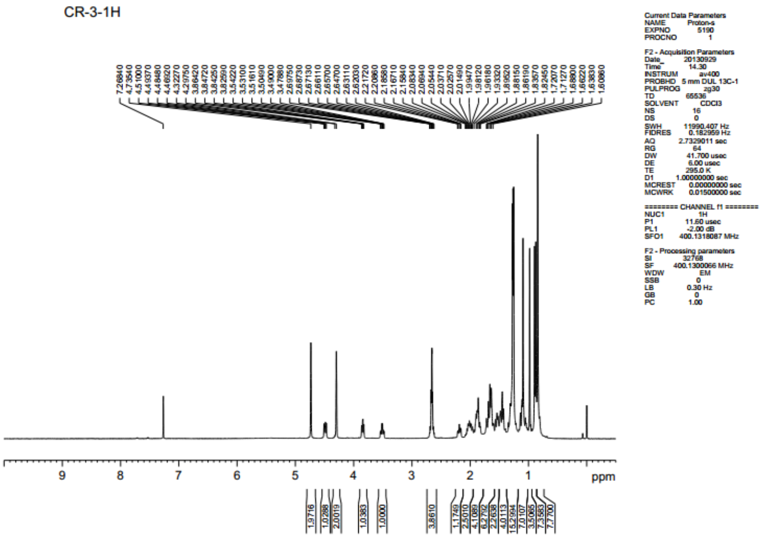

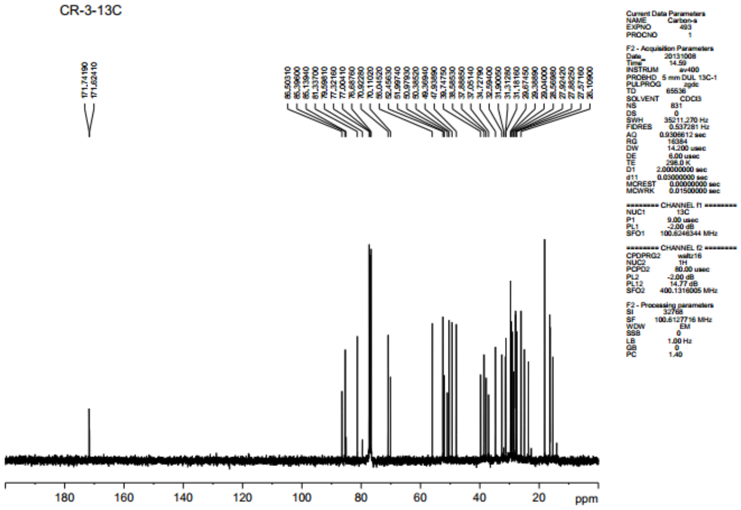

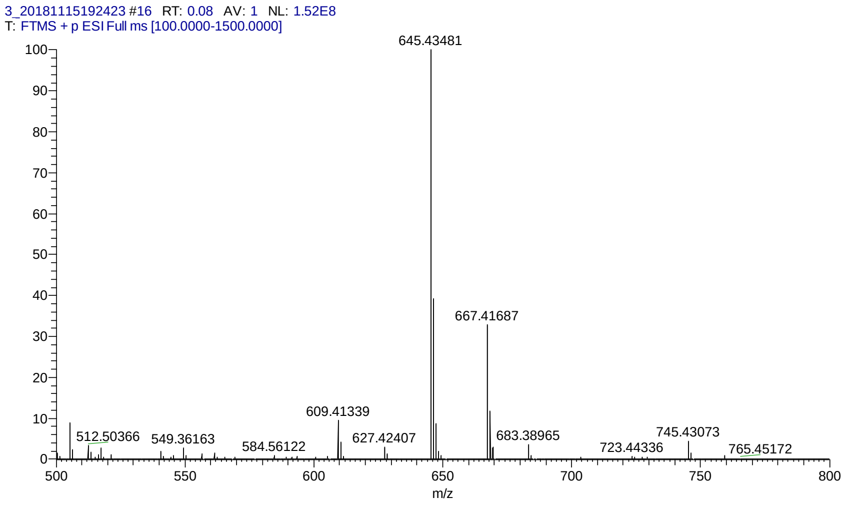

^{1}H NMR, ^{13}C NMR and HR-MS (ESI) spectra of compound **11**

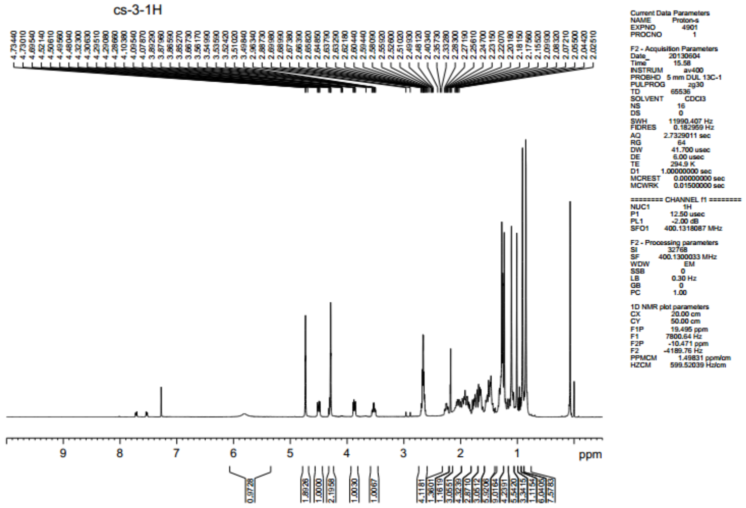

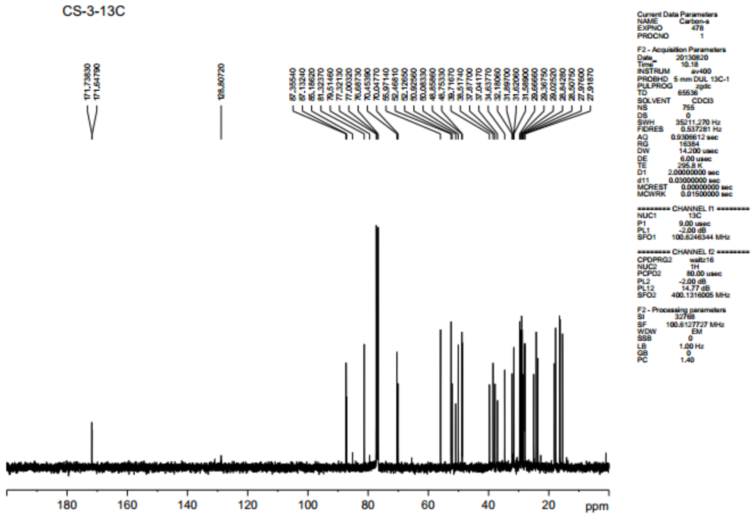

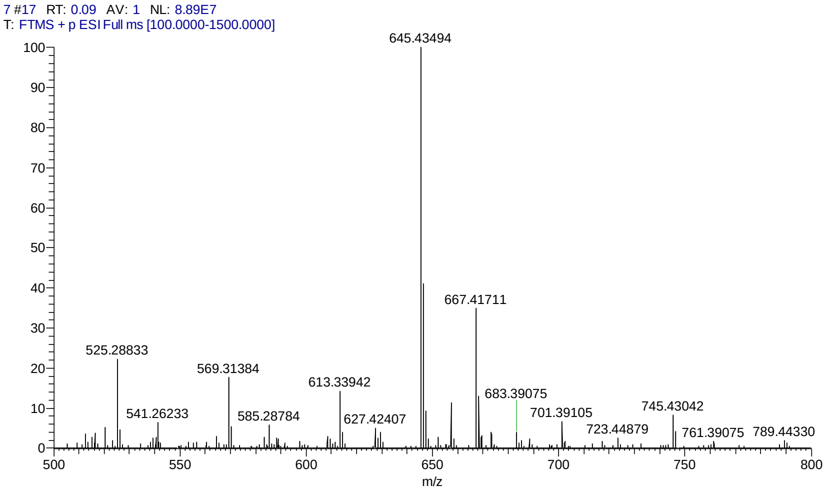

^1H NMR, ^{13}C NMR and HR-MS (ESI) spectra of compound **12**

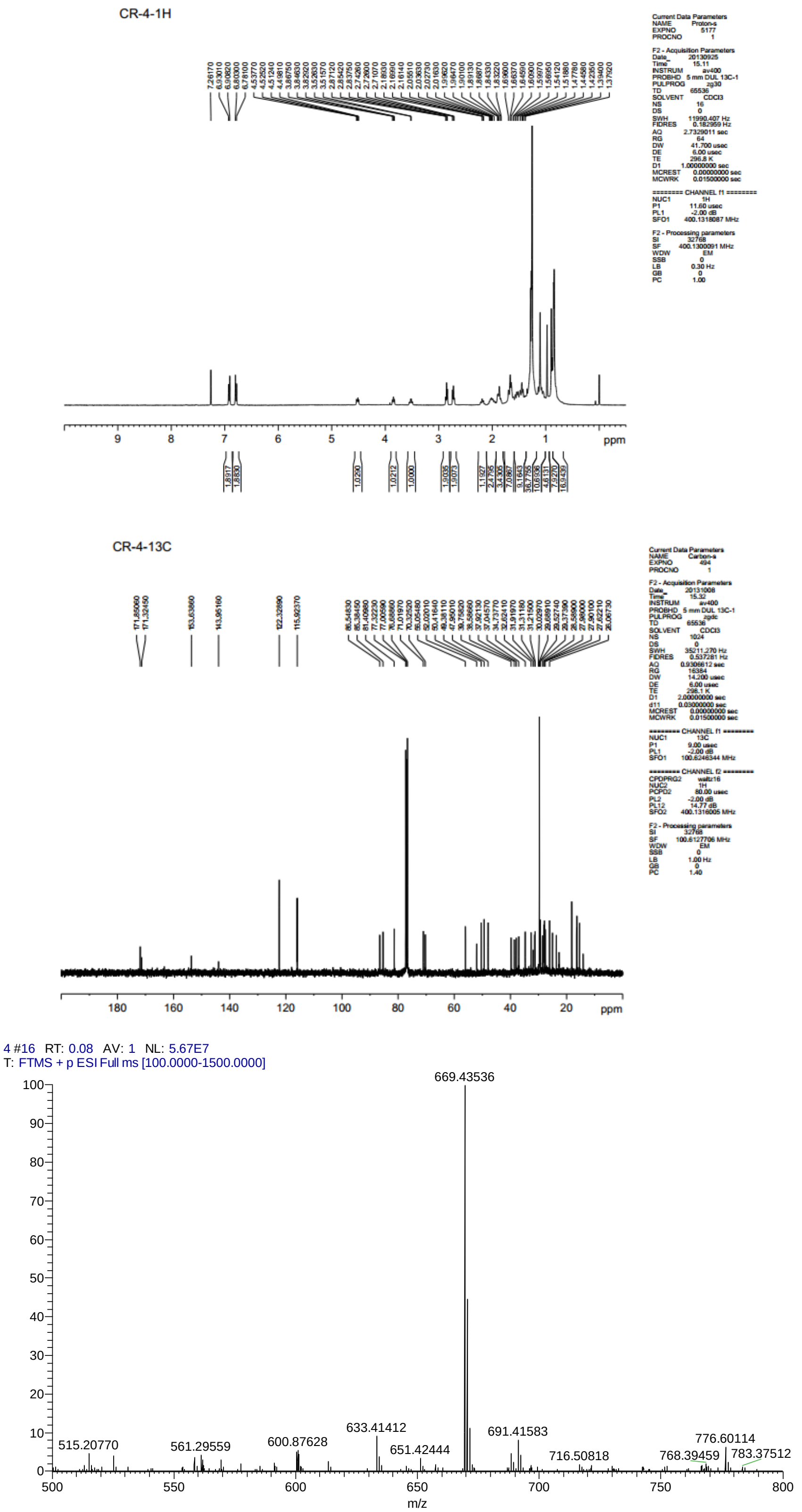

^{1}H NMR, ^{13}C NMR and HR-MS (ESI) spectra of compound **13**

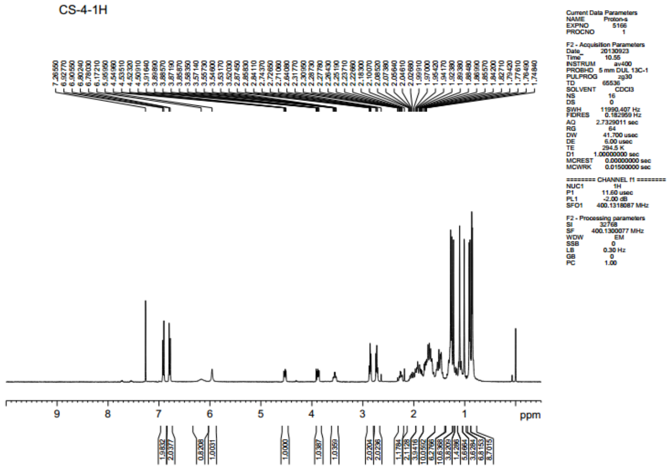

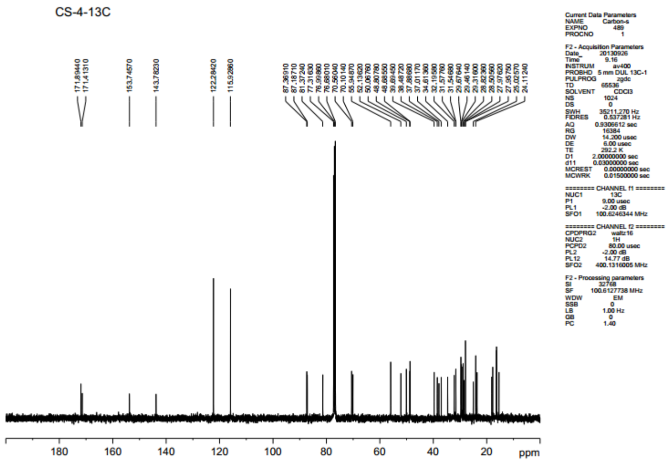

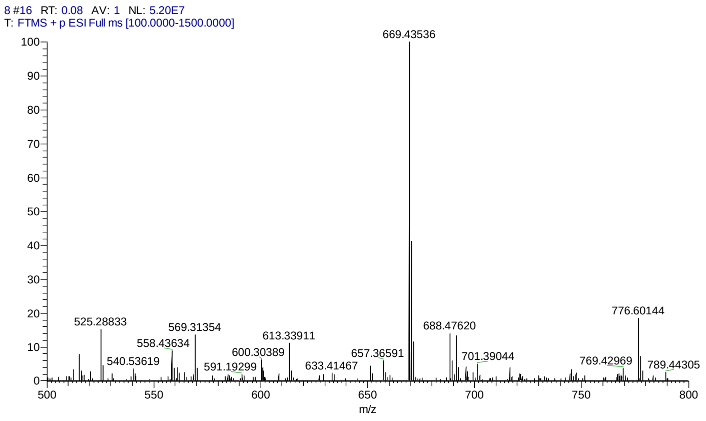

^{1}H NMR, ^{13}C NMR and HR-MS (ESI) spectra of compound **14**

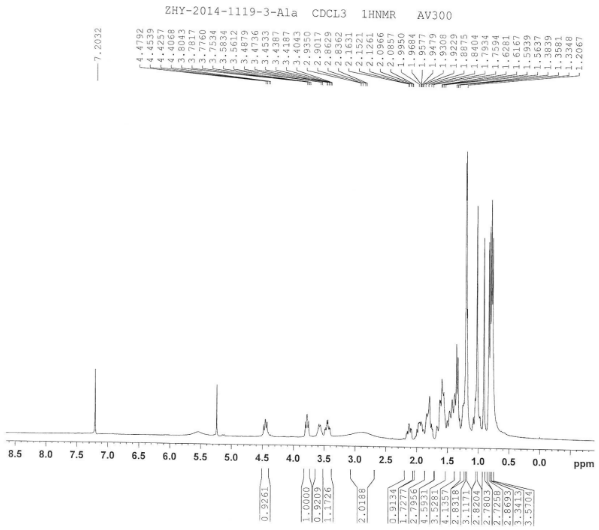

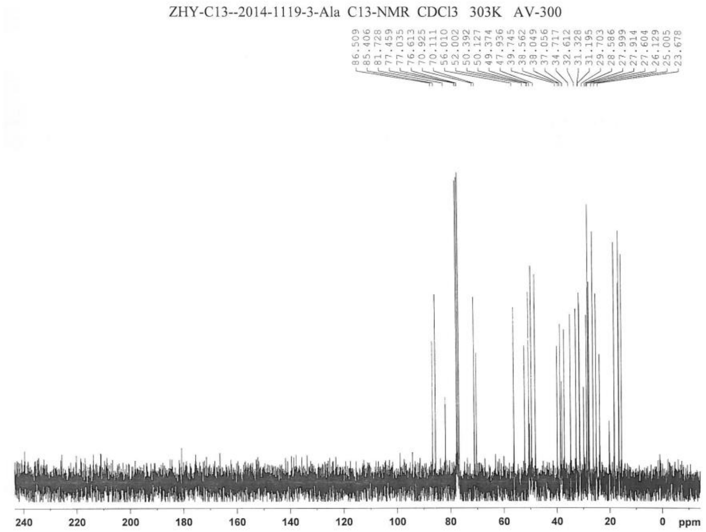

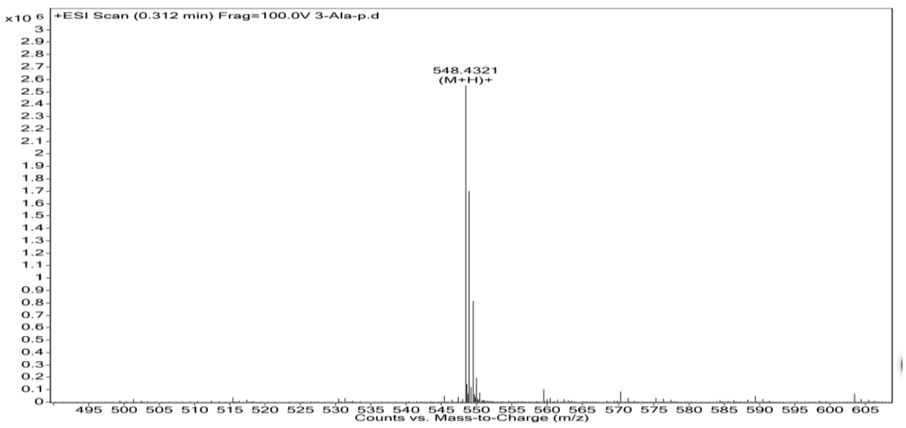

^{1}H NMR, ^{13}C NMR and HR-MS (ESI) spectra of compound **15**

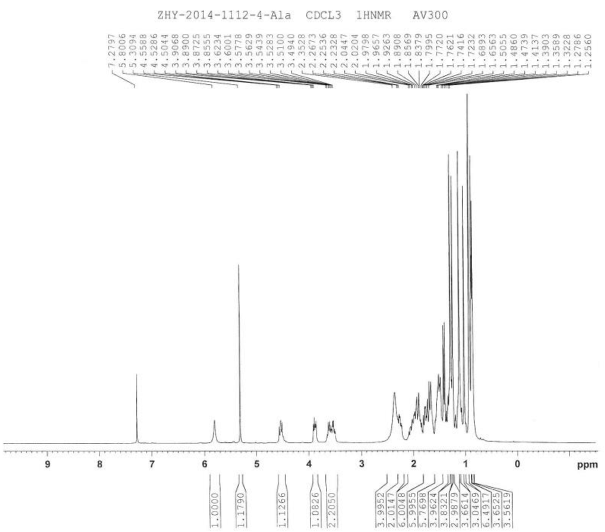

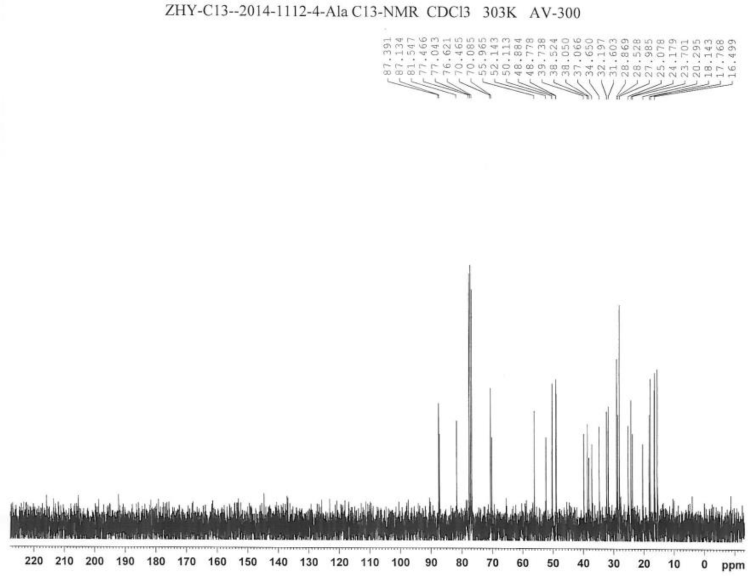

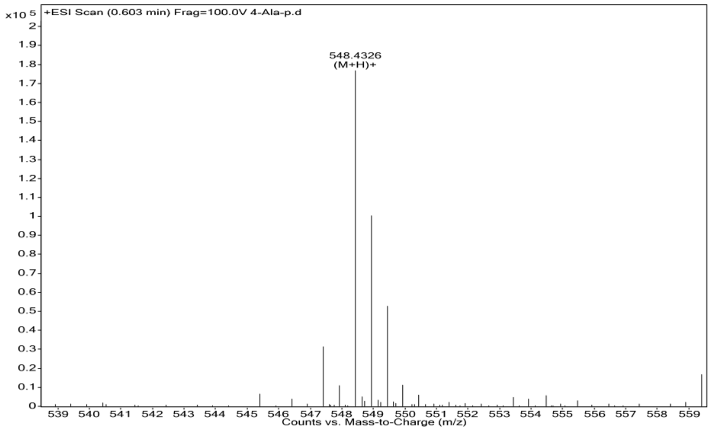

^{1}H NMR, ^{13}C NMR and HR-MS (ESI) spectra of compound **16**

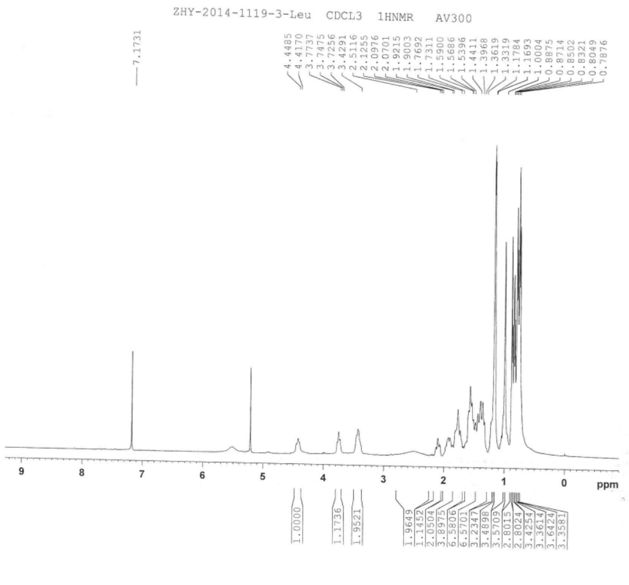

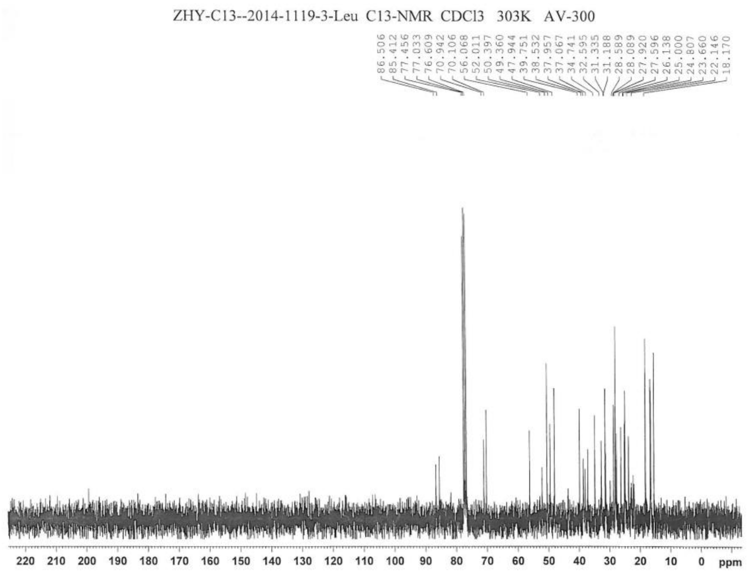

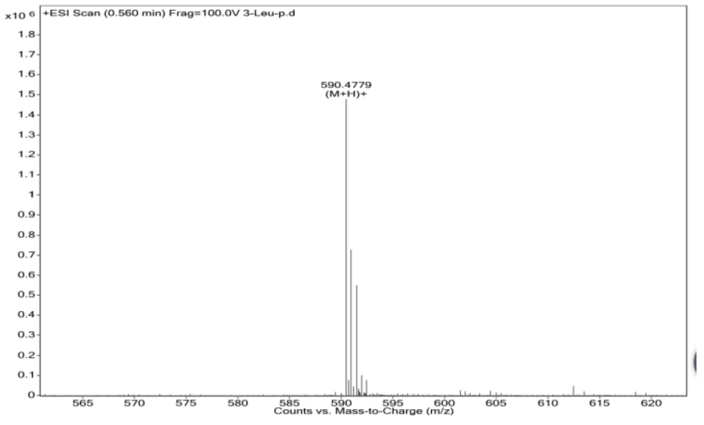

^{1}H NMR, ^{13}C NMR and HR-MS (ESI) spectra of compound **17**

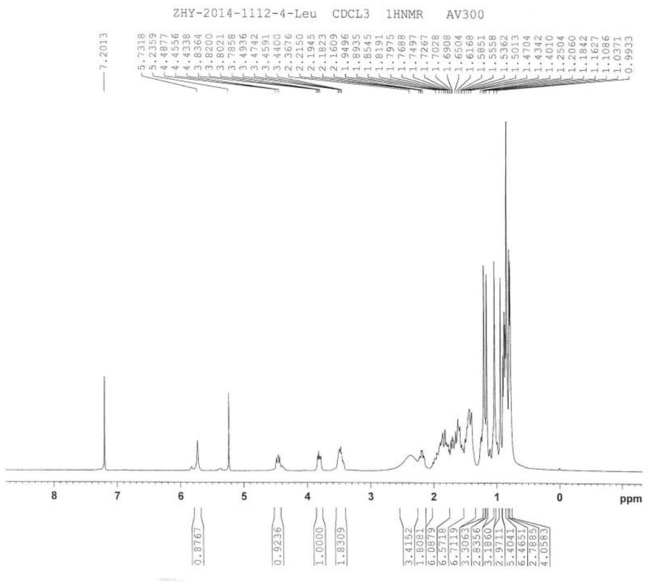

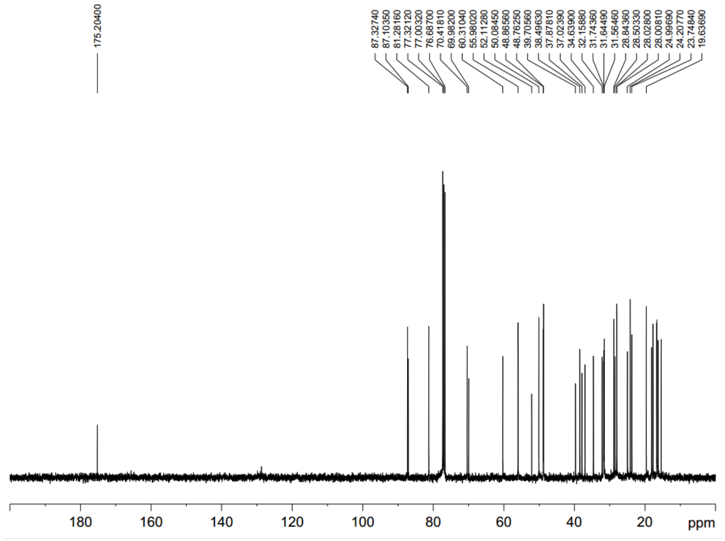

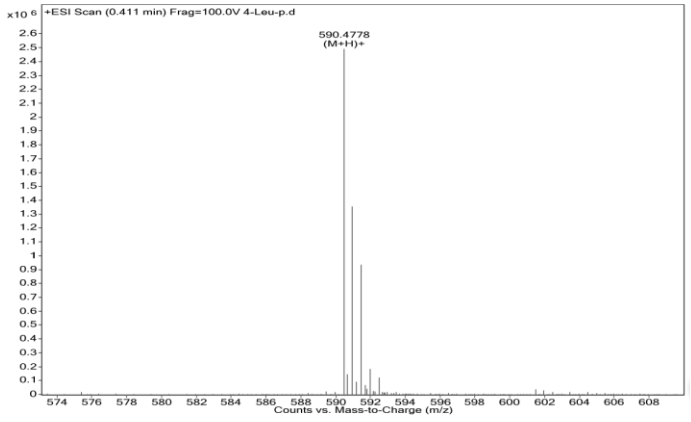

^{1}H NMR, ^{13}C NMR and HR-MS (ESI) spectra of compound **18**

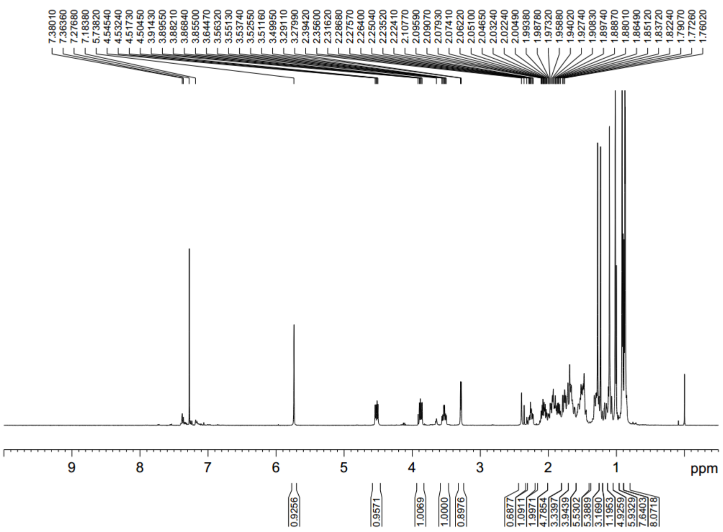

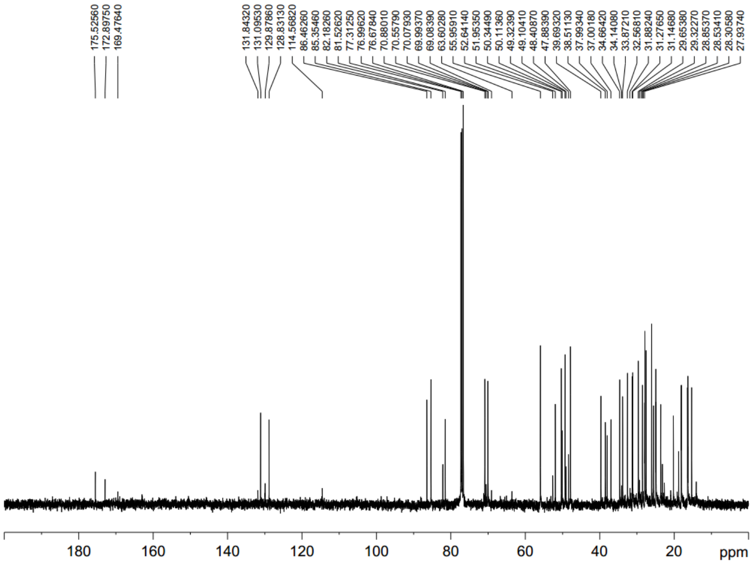

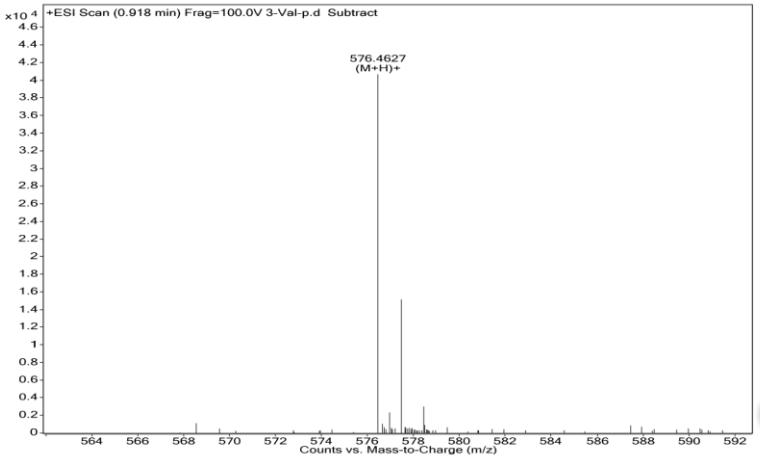

^{1}H NMR, ^{13}C NMR and HR-MS (ESI) spectra of compound **19**

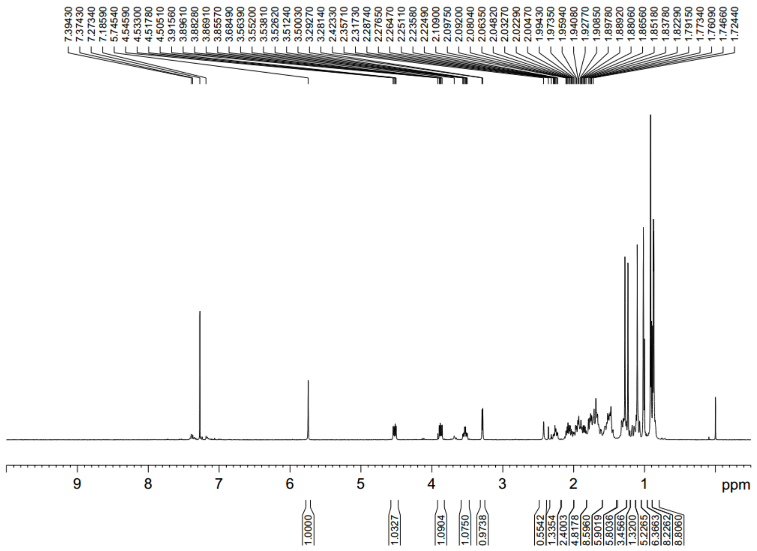

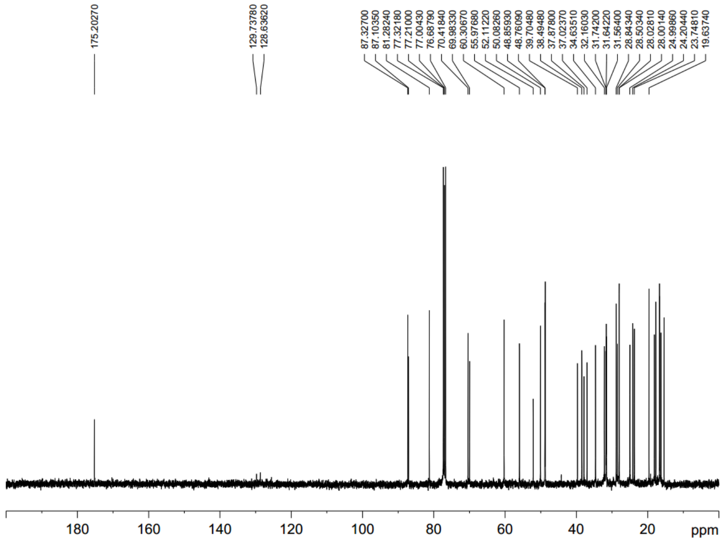

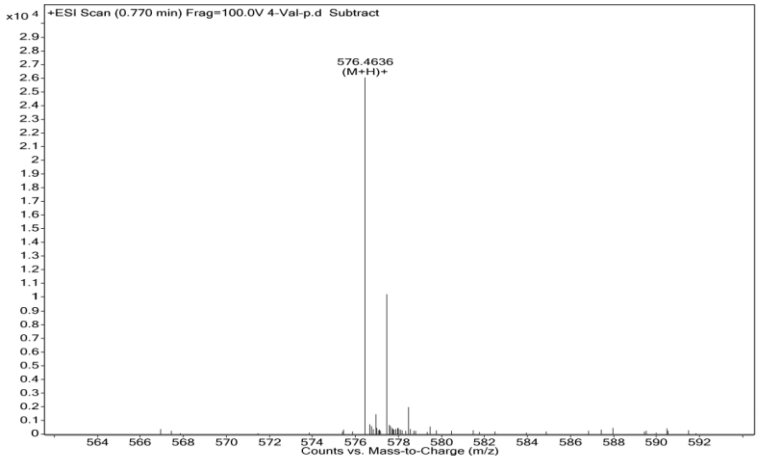

^{1}H NMR, ^{13}C NMR and HR-MS (ESI) spectra of compound **20**

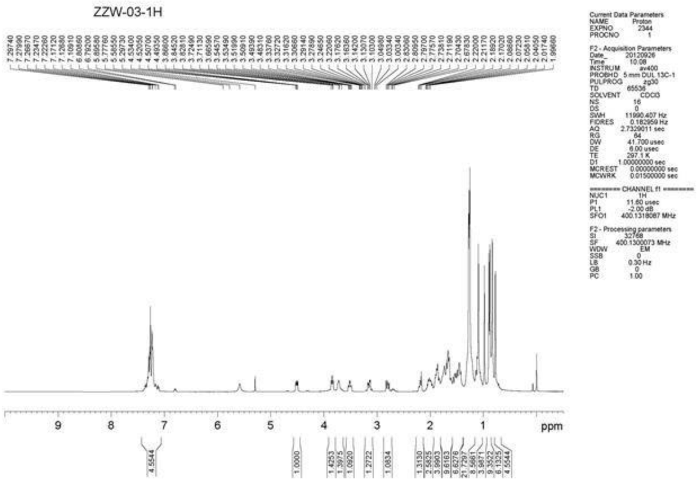

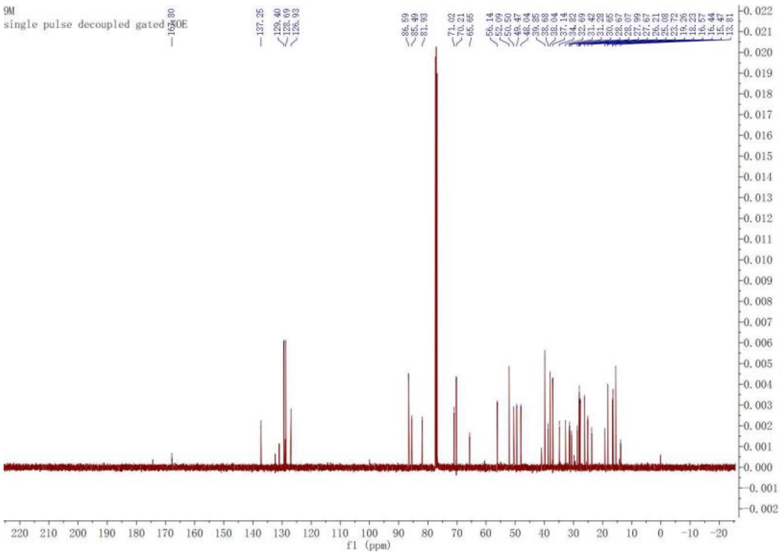

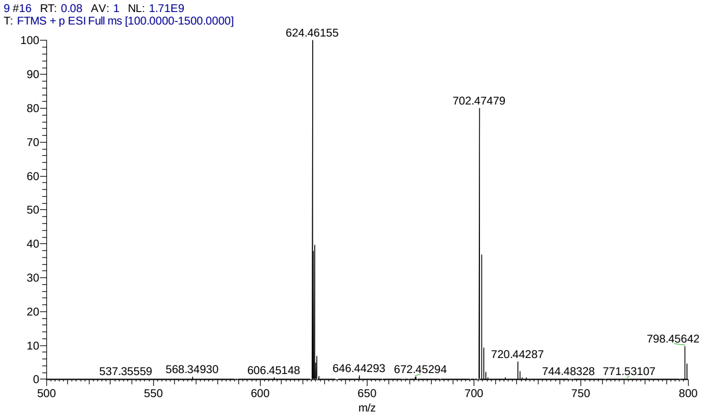

^{1}H NMR, ^{13}C NMR and HR-MS (ESI) spectra of compound **21**

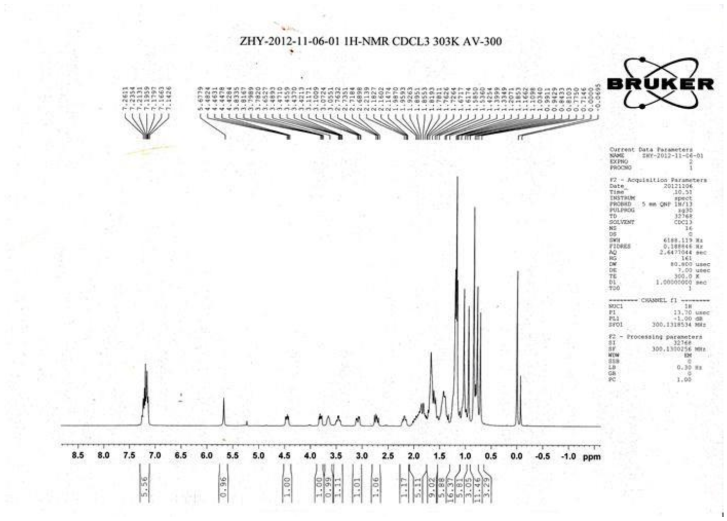

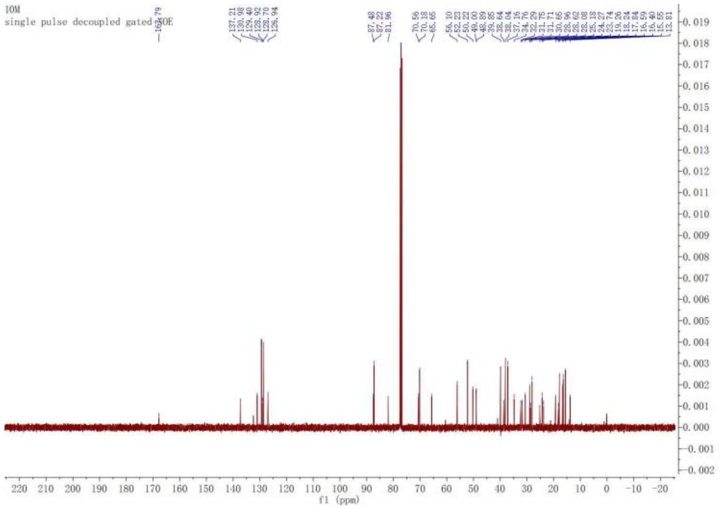

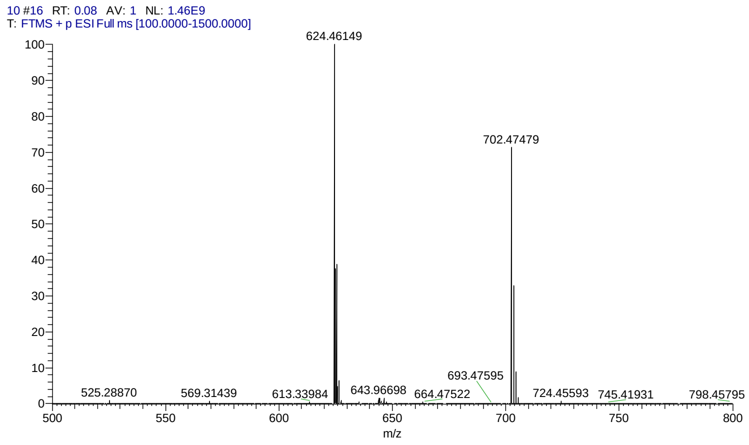

^{1}H NMR, ^{13}C NMR and HR-MS (ESI) spectra of compound **22**

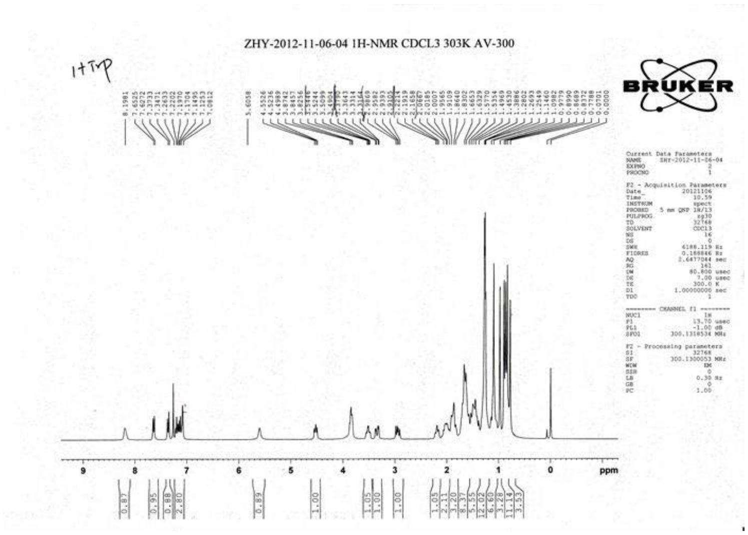

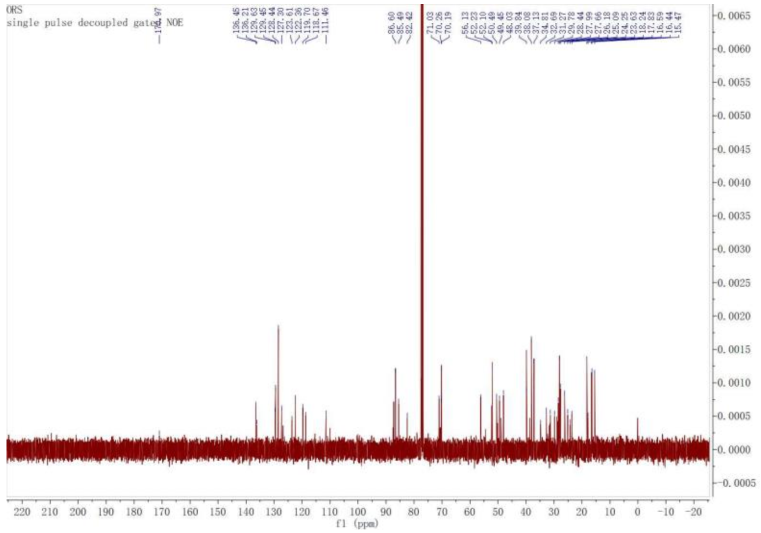

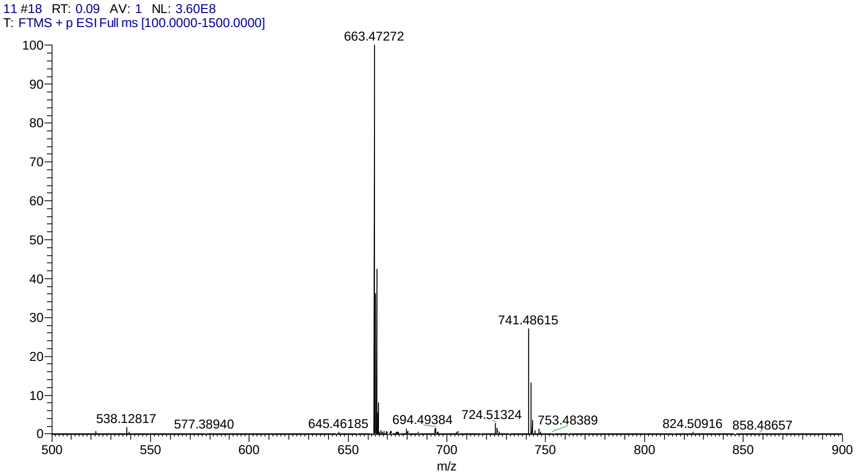

^{1}H NMR, ^{13}C NMR and HR-MS (ESI) spectra of compound **23**

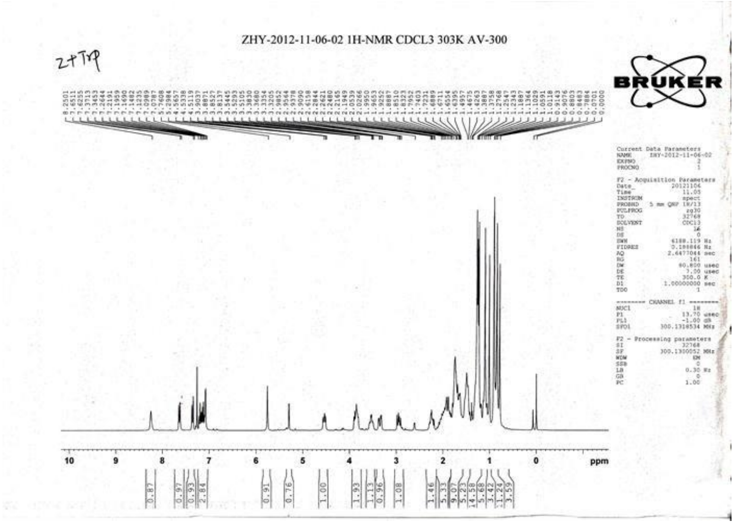

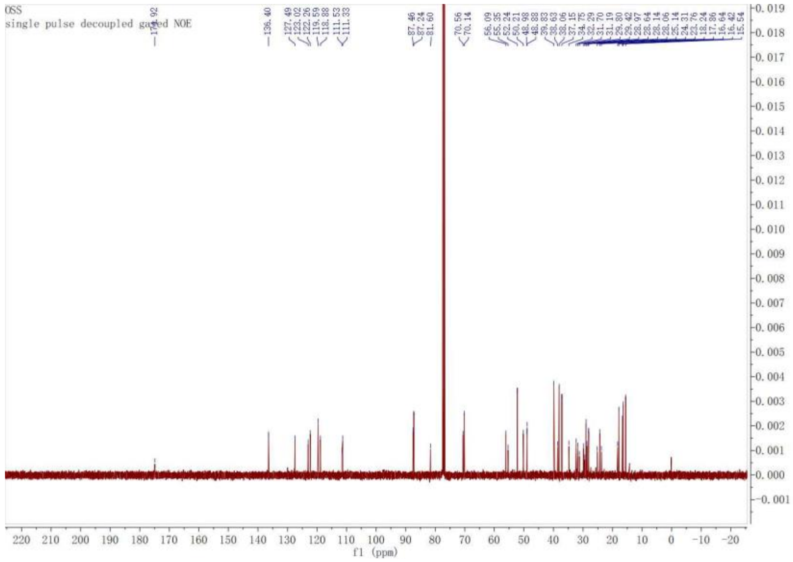

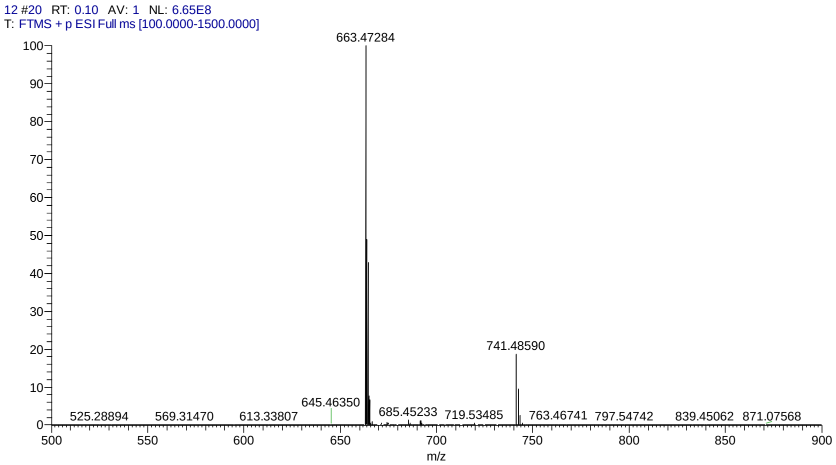

^{1}H NMR, ^{13}C NMR and HR-MS (ESI) spectra of compound **24**

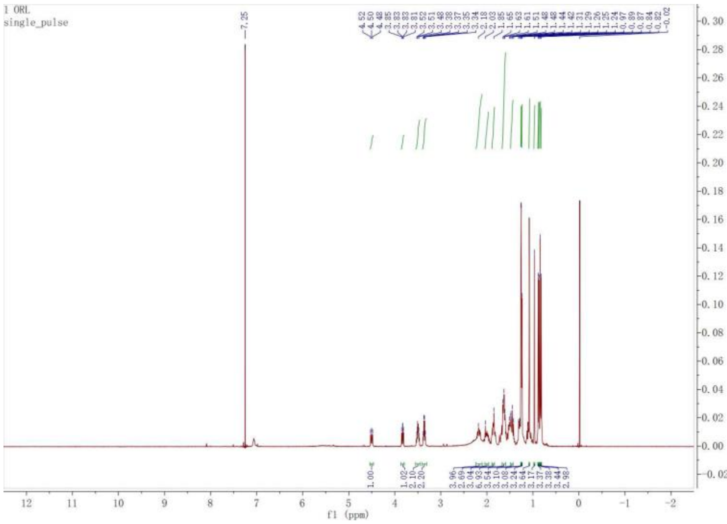

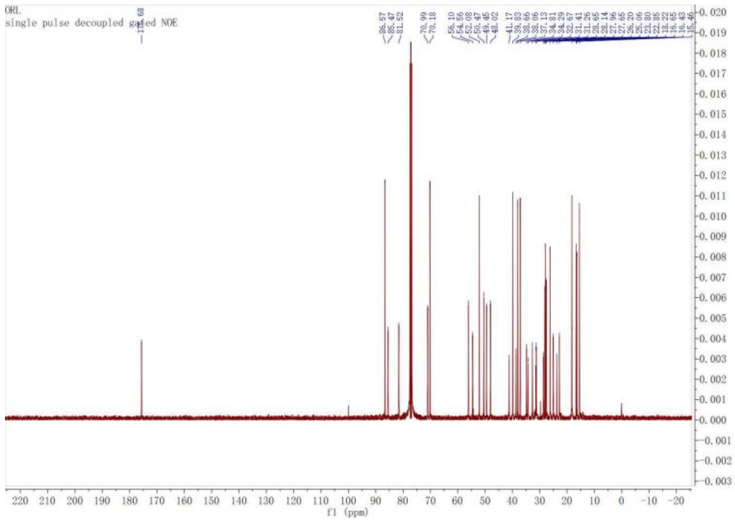

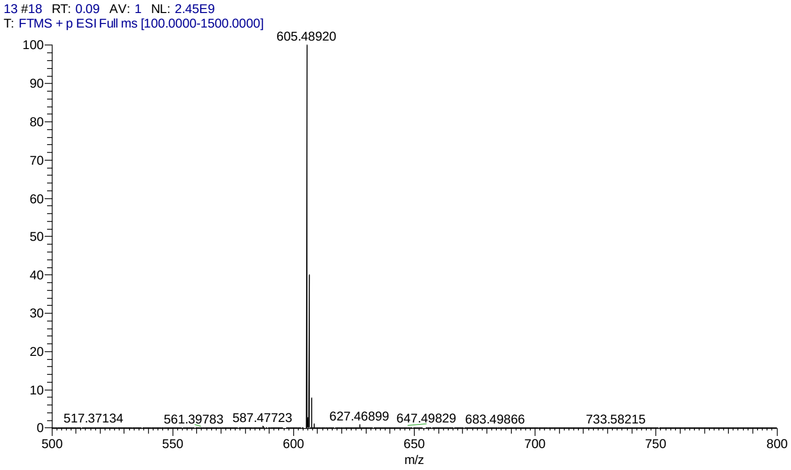

^1H NMR, ^{13}C NMR and HR-MS (ESI) spectra of compound **25**

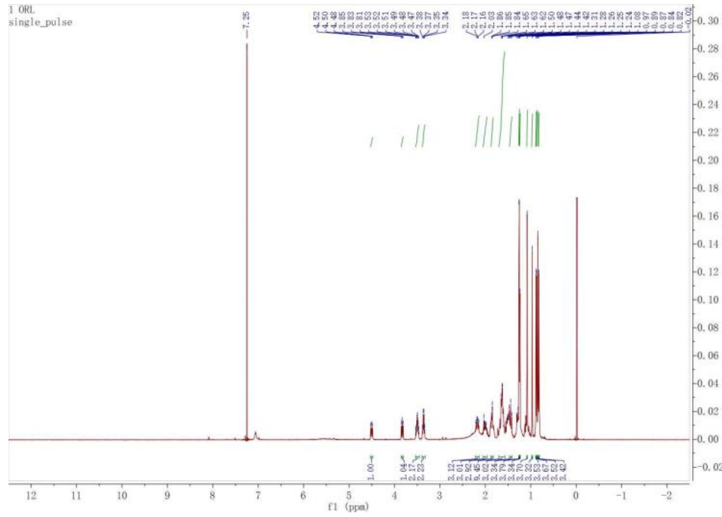

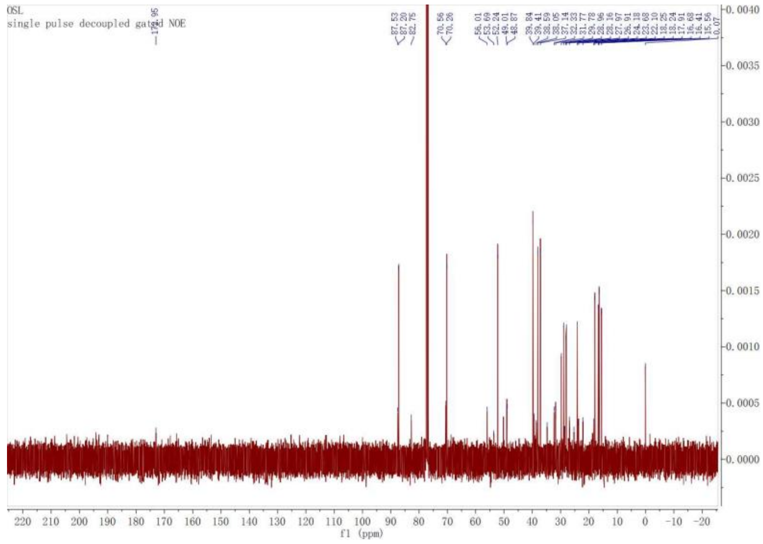

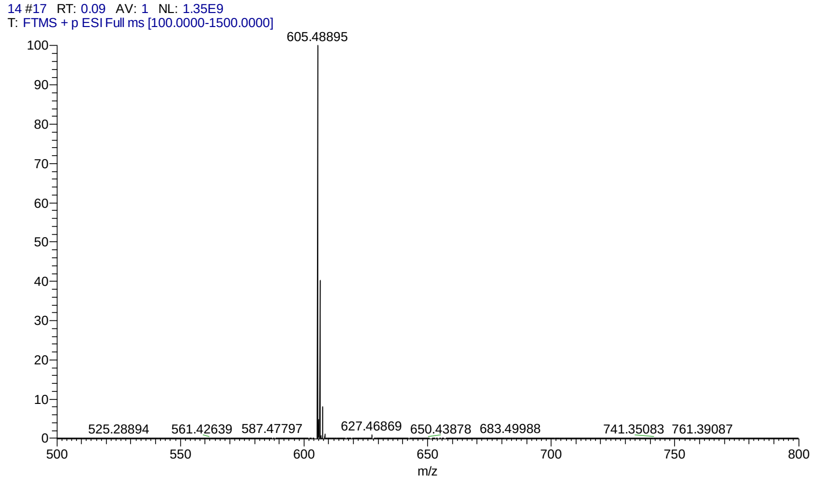

^{1}H NMR, ^{13}C NMR and HR-MS (ESI) spectra of compound **26**
